# Final weight prediction from body measurements in Kıvırcık lambs using data mining algorithms

**DOI:** 10.5194/aab-68-325-2025

**Published:** 2025-05-21

**Authors:** Ömer Şengül, Şenol Çelik

**Affiliations:** 1 Faculty of Agriculture Department of Animal Science, Bursa Uludağ University, 16000, Bursa, Türkiye; 2 Faculty of Agriculture Department of Animal Science, Bingöl University, 12000, Bingöl, Türkiye

## Abstract

This study was carried out to determine the final weight estimation of Kıvırcık lambs using body measurements via Chi-square automatic interaction detection (CHAID), exhaustive CHAID, classification and regression tree (CART), random forest (RF), multivariate adaptive regression spline (MARS), and bootstrap-aggregating multivariate adaptive regression spline (Bagging MARS) algorithms. For this purpose, height at withers (HW), back height (BH), croup height (CH), chest depth (CD), body length (BL), chest width (CW), and chest circumference (CC) were measured in the lambs. The statistical performances of these algorithms (CHAID, exhaustive CHAID, CART, RF, MARS, and Bagging MARS) were tested by using several goodness-of-fit criteria, namely the coefficient of determination ( $R^{2}=0.699$ , 0.699, 0.722, 0.662, 0.792, and 0.624), adjusted coefficient of determination (Adj. $R^{2}=0.633$ , 0.633, 0.721, 0.637, 0.768, and 0.609), coefficient of variation (CV % = 6.35 and 5.14, P<0.01 ), mean square error (MSE = 3.296, 3.296, 2.904, 4.461, 2.277, and 4.121), root mean square error (RMSE = 1.815, 1.815, 1.704, 2.112, 1.509, and 2.030), mean absolute error (MAE = 1.409, 1.409, 1.279, 1.702, 1.193, and 1.628), and mean absolute percentage error (MAPE = 3.925, 3.925, 3.578, 4.002, 3.335, and 3.967), between actual and predicted values of live body weight. With this, the best-fitted MARS model was chosen using cross-validation and user-defined parameter optimization. As a result, it has been shown that it is possible to make a successful estimation of the live weights of lambs by using some of the morphological features of the lambs.

## 1 Introduction

Body measurements obtained by measuring animal body parts are an important criterion in estimating body weights at later ages or at the end of the fattening period (El Khidir, 1980). Body weight is one of the most important performance indicators of animals and has the largest share in the selection of animals. The method generally applied in determining the live weights of animals is to weigh them with a scale suitable for this purpose. However, this situation poses a significant problem, especially in rural areas where animals cannot be weighed. The lack of weighing scales in small-scale livestock farms in rural areas creates difficulties for producers in determining live weights. For this purpose, animal owners evaluate their animals in terms of live weight based only on their appearance. This situation causes significant mistakes and, thus, economic losses for the producers (Slippers et al., 2000).

However, growth in animals is a complex biological process caused by different rates of development of body tissues. For this purpose, different methods have been developed to predict the development of the body's musculoskeletal system in animals, and the relationships between external measurements of the body and live weights have been investigated (Atta and EL Khidir, 2004). Analysis of the fattening-period growth performance of animals allowed for the determination of the most appropriate feeding strategies for them and for the determination of the optimum slaughter age (Souza et al., 2013). Using body measurements will provide important benefits to producers in terms of increasing their income from sales of livestock. Follow-up on growth using body measurements will allow for the obtainment of the optimum fattening weight. Noor et al. (2001) and Abegaz et al. (2010) reported that a slow growth rate in animals causes a low market weight, which is one of the most important factors limiting profitability. Kunene et al. (2009) measured body weight and wither heights in Zulu sheep of different ages and explained that age and gender are the most important factors causing changes in body weight.

This study was conducted on Kıvırcık lambs raised for fattening purposes. The Kıvırcık breed originated in Türkiye, and it is a combined productive breed raised mostly in the Thrace and southern Marmara regions. The Kıvırcık breed constitutes approximately 6 % to 7 % of the total sheep population in Türkiye (Öner et al., 2014). Due to its high meat quality, it is mainly used for meat production.

Data mining algorithms can be used as an effective parameter in the genetic and phenotypic identification of sheep breed characteristics (Hamadani et al., 2022). The data obtained by estimating the live weight with these methods are important for sheep producers. In studies on this subject, the importance of tree-based algorithms has not been sufficiently emphasized (Eyduran et al., 2008; Yakubu, 2012). In Pakistan, live-weight estimates were made in Harnai and Baluchi sheep using different data mining algorithms (Ali et al., 2015; Mohammad et al., 2012). Karabacak et al. (2017) estimated live weight in sheep using various body measurements with Chi-square automatic interaction detection (CHAID), exhaustive CHAID, and classification and regression tree (CART) data mining algorithms and reported that the CHAID algorithm gave better results than the others. Çakmakçi (2022) performed body weight estimation in Norduz sheep using data mining and machine learning algorithms. In the aforementioned study, the prediction performance validated using the test dataset displayed that the random forest algorithm outperformed model average neural networks, support vector machines with a radial basis function kernel, and CART models, with the lowest values in terms of mean absolute error, root mean squared error, and mean absolute percent error. Ghotbaldini et al. (2019) used a multilayer perceptron to determine the breeding values for body weight at 6 months of age in Kermani sheep. The results indicated that the multilayer perceptron with seven input variables and seven neurons in the hidden layer, with a correlation coefficient of 0.703, and the multilayer perceptron with nine input variables and seven neurons in the hidden layer, with a correlation coefficient of 0.864, were both capable of predicting the breeding values for body weight at 6 months of age in Kermani sheep. In the study, it was emphasized that both have the ability to predict breeding values for live weight in Kermani sheep at 6 months of age.

In the study of Abbas et al. (2021), CHAID, exhaustive CHAID, CART, and artificial neural network (ANN) methods were used to determine the live weight of Thalli sheep. The researchers reported that, among the algorithms used, CHAID provided the most appropriate estimation ability in the estimation of live weight for Thalli ewes. In the study, overall, the applied algorithms and standards accurately estimated body weight and so can assist in deciding the amount of feed required for the animals.

In a more effective specification of the studied data, the best algorithm selection is of the essence for sheep breeding. For this reason, the main targets of the present research were to measure the performance of CART, random forest, multivariate adaptive regression spline (MARS), and bootstrap-aggregating multivariate adaptive regression spline (Bagging MARS) data mining algorithms fitted in order to estimate final body weight (SW) from several body measurements (height at withers (HW), back height (BH), croup height (CH), chest depth (CD), body length (BL), chest width (CW), and chest circumference (CC)) of Kıvırcık lambs and, in particular, to display how to interpret the results taken from the research.

## 2 Material and methods

In this study, the treatment of lambs was carried out in a semi-open barn in a sheep farm belonging to the Agricultural Application and Research Center of Uludağ University. In the study, 40 Kıvırcık male lambs aged 2.5–3 months were used as animal material. The live weights of the lambs included in the experiment varied between 23–25 kg. The study was carried out in the period of April–June 2022 over the course of 42 d. The animals were fed with concentrated feed and alfalfa hay during the fattening period. The lambs were fed with concentrated feed ad libitum, and, additionally, 200 g of alfalfa hay per day was given. The nutrient compositions of the concentrated feed and alfalfa used are given in Table 1, and the content of the concentrated feed is given in Table 2.

**Table 1:** Nutrient composition of concentrated feed and alfalfa used in the experiment.

Nutritional content (%)	Concentrated feed	Alfalfa
Dry matter	86.42	90.75
Crude oil	0.87	3.23
Crude cellulose	10.91	21.78
Crude ash	8.56	9.77
Cellulose	9.70	21.82
Hemicellulose	11.52	11.51
Nitrogen-free core substances	57.27	45.67
Neutral detergent fiber (NDF)	26.83	37.27
Acid detergent fiber (ADF)	15.31	25.76
Acid detergent lignin (ADL)	5.61	5.91
Crude protein	22.40	19.55
ME kcal per kilogram dry matter	2310	2055

**Table 2:** The composition of the concentrated feed.

Raw materials	Ratio (%)
Barley	73.0
Sunflower seed meal	25.0
Marble powder (CaCO_3_)	1.4
Salt	0.5
Mineral–vitamin mixture^*^	0.1

^*^Per kilogram: vitamin A 300 000 IU, vitamin D_3_ 50 000 IU, 1250 mg of vitamin E, 3000 mg of manganese (oxide), 3000 mg of iron (sulfate), 4500 mg of zinc (oxide), 1000 mg of copper (sulfate), 30 mg of cobalt (mono carbonate), 45 mg of iodine (calcium iodate), 12 mg of selenium selenite, and 969 066 mg of filler (razmol or Ca CO_3_).

On the 42nd day of the treatment, the final weights and body measurements of the lambs were determined. A measuring stick, measuring caliper, and measuring tape were used to determine body measurements. The properties were measured as follows:

- Height at withers is the height from the top of withers to the ground.
- Back height is the height from the dorsal vertebrae to the ground.
- Croup height is the height from the top of the croup to the ground.
- Chest depth is the depth from the withers to the sternum.
- Body length is the length from the anterior edge of shoulder to the posterior edge of the ischium.
- Chest width extends to behind the shoulder.
- Chest circumference extends to behind the posterior edge of the shoulders at the point of least perimeter.

## 3 Statistical analysis

The values of the body measurements obtained were analyzed with classification and regression tree (CART), random forest (RF), multivariate adaptive regression spline (MARS), and bootstrap-aggregating multivariate adaptive regression spline (Bagging MARS) algorithms. With the intention of predicting final weight from the selected explanatory variables, classification and regression tree (CART), multivariate adaptive regression spline (MARS), Bagging MARS, and random forest (RF) data mining algorithms were implemented in the current study.

The CART algorithm uses classification and regression trees (Breiman et al., 1984). By splitting a subset into two smaller subsets based on the time, homogeneous subsets are reached in the tree; CART (classification and regression tree) recursively generates a binary regression tree (Ali et al., 2015; Akin et al., 2016). The splits are perceived using the twoing criterion, and the acquired tree is pruned by cost–complexity pruning. When CART is used, misclassification in tree induction can take into account the costs. It also makes it possible for users to generate prior probability distributions in advance. Creating regression trees is an important property of CART. Regression trees are trees whose leaves do not have a class but predicted a real number (El Seddawy et al., 2013).

Versatile node separation is available in CHAID (Chi-square automatic interaction detection) and exhaustive CHAID. Corrected significance values are acquired using the Bonferroni method, combining and splitting criteria (Ali et al., 2015). In the event that the Chi-square tests yield significant results, the CHAID method is utilized to choose a criterion variable and to choose a number of independent-variable categories. The process then moves on to node construction and segment configuration, which end when there is no discernible link between the explanatory factors and the criteria. The first node contains the most important independent variable. Nonetheless, compared to other non-criterion techniques like cluster analysis, CHAID exhibits higher efficiency in terms of the quantity of data and the number of variables (Kass, 1980). The rule of thumb, also known as the stopping rule, is crucial when using the CHAID algorithm to determine how big of a tree should develop (Milanovic and Stamenkovic, 2016). The CHAID decision tree method was created by Biggs et al. (1991) and was updated to create the exhaustive CHAID algorithm in order to address some of its shortcomings. Exhaustive CHAID varies from CHAID in that it looks at every split option on each node and continues splitting even after the ideal split is achieved. The predictor variable's categories are continuously combined until there are just two remaining subcategories. The process consists of three main steps: splitting, merging, and halting (Novita et al., 2015). The minimum number of animals for parent and child nodes was set to be 10:5 to create an optimal regression tree with more nodes. The measurement of intra-node variance in a decision tree created with any data mining algorithm, i.e., risk estimation, shows the prediction accuracy of the decision tree.

MARS (multivariate adaptive regression spline) is a data mining method that uses a set of segmented linear or cubic segments created to simulate nonlinear connections between dependent variables (splines) and inputs. In each subspace, the MARS algorithm obeys a spline function called the basis function (BF) and splits the space of the input parameters into different subspaces. Knots represent the end of one data field and the beginning of another. The MARS algorithm tries to detect all possible interactions between the variables by controlling for all the degrees of interaction. The method can also track hidden connections and complex structures seen in data points in a high-dimensional dataset. It is expressed as the MARS model (Zhang and Goh, 2016):

1 $$f\left(x\right)=\beta_{0}+\sum_{m=1}^{m}\beta_{m}\lambda_{m}\left(x\right),$$

where f(x) is the expected response, $\beta_{0}$ and $\beta_{m}$ are parameters that are calculated to give the best data fit, and m is the number of BFs in the model. The basis function that makes up the MARS model is derived from a single variable spline function or a combination of multiple spline functions for various predictive inputs. The spline BF, $\lambda_{m}(x)$ , is described as follows:

2 $$\lambda_{m}\left(x\right)=\prod_{k=1}^{k_{m}}\left[s_{km}\left(X_{v\left(k,m\right)}-t_{k,m}\right)\right],$$

where $t_{k,m}$ denotes the knot location; $s_{km}$ denotes the right and/or left regions of the corresponding step function, taking either 1 or - 1; $v\left(k,m\right)$ denotes the predictor variable's label; and $k_{m}$ is the number of knots. The MARS algorithm generates BFs and further overfits the data by examining a large number of BFs. To avoid overfitting, duplicate BFs are subtracted from Eq. (1) backwards. In order to get rid of duplicate BFs in the MARS algorithm, generalized cross-validation (GCV) is performed. The GCV is as follows (Kornacki and Cwik, 2005):

3 $$\mathrm{GCV}=\frac{\frac{1}{N}\sum_{i=1}^{n}{\left[y_{i}-f^{\prime }(x_{i})\right]}^{2}}{{\left[1-\frac{C(B)}{N}\right]}^{2}}.$$

Here, N is the total number of points in the data. C(B) indicates a complexity penalty that increases in increments with the number of BFs in the method (Naser et al., 2022).

4 C(B)=(B+1)+d(B)

In MARS modeling, resampling was handled by 10-fold cross-validation.

The Bagging MARS model evolved from an ensemble of MARS models (Chen et al., 2020). The Bagging estimator (Breiman, 1996) is the expectancy of an ensemble of models; that is,

5 $${\hat{f}}_{\text{Bagging}}=E\left[\hat{f}\left(x\right)\right].$$

The Bagging MARS algorithm might be approximated by applying the Monte Carlo technique:

6 $${\hat{f}}_{\text{Bagging}}\approx \frac{1}{B}\sum_{b=1}^{B}{\hat{f}}_{b}\left(x\right).$$

The accuracy value of the Monte Carlo technique is determined depending on the constant B , which is determined according to the sample size and the current computational cost. Therefore, the number of bootstrap samples was set to be 5.

The random forest (RF) algorithm is an ensemble classifier implemented to improve accuracy. RF is composed of many decision trees. The classification error of RF is lower than that of other classic classification algorithms (Farnaaz and Jabbar, 2016). In RF, all variables are considered to be predictors, and total tree height is used as a response variable (Yang et al., 2022). The “ranger” function was used to implement random forests faster and more memory efficiently in order to analyze data compared to other random forest packages commonly applied in R software (Wright and Ziegler, 2017).

When working with MARS, Bagging MARS, RF, CHAID, exhaustive CHAID, and CART methods, no assumption is made regarding the distribution of the data to be evaluated. Thus, these methods can be used for ordinal, nominal, and continuous outcome variables.

In order to compare the predictive performances of the CART, RF, MARS, and Bagging MARS algorithms in the 10-fold cross-validation, the following model evaluation criteria were calculated (Willmott and Matsuura, 2005; Liddle, 2007; Chen and Li, 2014; Chen and Guestrin, 2016).

The coefficient of determination is calculated as follows:

7 $$R^{2}=1-\frac{\sum_{i=1}^{n}(Y_{i}-{\hat{Y}}_{i}{)}^{2}}{\sum_{i=1}^{n}(Y_{i}-\bar{Y}{)}^{2}}.$$

The adjusted coefficient of determination is calculated as follows:

8 $$\text{Adj}.R^{2}=1-\frac{\frac{1}{n-k-1}\sum_{i=1}^{n}{\left(Y_{i}-{\hat{Y}}_{i}\right)}^{2}}{\frac{1}{n-1}\sum_{i=1}^{n}{\left(Y_{i}-\bar{Y}\right)}^{2}}.$$

The mean square error is calculated as follows:

9 $$\mathrm{MSE}=\frac{1}{n}\sum_{i=1}^{n}(Y_{i}-{\hat{Y}}_{i}{)}^{2}.$$

The root mean square error (RMSE) is expressed by the following formula:

10 $$\mathrm{RMSE}=\sqrt{\frac{1}{n}\sum_{i=1}^{n}(Y_{i}-{\hat{Y}}_{i}{)}^{2}}.$$

The mean absolute error (MAE) is the average absolute prediction error. It is less sensitive to outliers. The formula is as follows:

11 $$\mathrm{MAE}=\frac{1}{n}\sum_{i=1}^{n}\left|Y_{i}-{\hat{Y}}_{i}\right|.$$

The mean absolute percentage error (MAPE) is calculated as follows:

12 $$\mathrm{MAPE}=\frac{1}{n}\sum_{i=1}^{n}\left|\frac{Y_{i}-{\hat{Y}}_{i}}{Y_{i}}\right|.100.$$

R software was used for the analyses using 10-fold cross-validation (R Core Team, 2020). Results were obtained using the RF algorithm “randomForest” and the “earth” package of the MARS and Bagging MARS algorithms. Data mining algorithm model evaluation performance criteria were evaluated using the “ehaGoF” package (Eyduran, 2020).

Using the R software “corrplot” package, Pearson correlation coefficients between SW and body characteristics were calculated. Also, we tested for any multicollinearity problems between the independent variables at the outset of the analysis, and it was discovered that there were none. The CART algorithm was analyzed with the SPSS V. 26.0 package (2019).

## 4 Results

Descriptive statistics for the measurements of body characteristics in Kıvırcık lambs are given in Table 3.

**Table 3:** Descriptive statistics of the variables studied (body features) in the study.

	N	Min.	Max.	$\bar{X}$	SD	SE	CV (%)
HW	40	6.50	68.00	60.575	9.426	1.490	15.561
BH	40	50.00	67.00	59.563	3.576	0.565	6.004
CH	40	56.00	70.00	64.288	3.234	0.511	5.030
CD	40	24.00	32.50	27.888	1.876	0.297	6.727
BL	40	6.00	75.00	67.500	10.401	1.645	15.409
CW	40	14.00	18.00	15.775	1.086	0.172	6.884
CC	40	76.00	93.50	84.263	4.118	0.651	4.887
SW	40	28.36	44.36	35.762	3.354	0.530	9.379

HW denotes height at withers (cm), BH denotes back height (cm), CH denotes croup height (cm), CD denotes chest depth (cm), BL denotes body length (cm), CW denotes chest width (cm), CC denotes chest circumference (cm), and SW denotes final weight (kg). $\bar{X}$ is the Mean, SD is the standard deviation, and SE is the standard error.

According to the results given in Table 3, if the coefficient of variation of the measured parameters is less than 20 %, it is assumed that there is a homogeneous distribution among the individuals examined (Liu et al., 2020). A low coefficient of variation indicates that the standard deviation from the average data is low and that the reliability of the average data is high (Şahin, 2021).

Correlation coefficients between body characteristics are also presented in Table 4.

**Table 4:** Correlation coefficients of the relationship between variables.

	HW	BH	CH	CD	BL	CW	CC	SW
HW	1							
BH	0.239	1						
CH	0.308	0.862^***^	1					
CD	0.177	0.479^**^	0.565^***^	1				
BL	0.084	0.280	0.220	0.107	1			
CW	0.146	0.098	0.066	0.148	0.245	1		
CC	0.208	0.378	0.419^**^	0.618^***^	0.205	0.553^***^	1	
SW	0.187	0.530^***^	0.580^***^	0.630^***^	0.295	0.551^***^	0.784^***^	1

Note that ^*^ denotes P<0.05 , ^**^ denotes P<0.01 , and ^***^ denotes P<0.001 .

The Pearson correlation coefficients ( r ) between HW and BH, CH, CD, BL, CW, CC, and SW were positive and ranged from 0.084 to 0.308. However, these correlation coefficients were low. Highly significant ( P<0.01 and P<0.001 ) and stronger relationships ( r= 0.479–0.862) between BH, CH, CD, and SW were obtained among the lambs. Highly significant ( P<0.01 and P<0.001 ) and stronger relationships ( r= 0.419–0.580) between CH, CH, CD, and SW were obtained among the lambs. Highly significant ( P<0.001 ) and stronger relationships ( r=0.618 and 0.630) between CD, CC, and SW were obtained among the lambs. Lower correlation coefficients ( r= 0.205–0.295) were found for the BL, CW, CC, and body weight. High correlation coefficients ( r=0.551 and 0.553) were calculated for CW, CC, and SW and ( r=0.784 ) between CC and SW. In addition, goodness-of-fit statistics such as the coefficient of determination ( $R^{2}$ ), the adjusted $R^{2}$ (Adj. $R^{2}$ ), mean square error (MSE), root mean square error (RMSE), mean absolute error (MAE), and mean absolute percentage error (MAPE) were calculated to obtain the best estimate for the statistical methods used to estimate the dependent variable (SW). A summary of the results of the random forest (RF), MARS, Bagging MARS, and CART algorithms in terms of the predictive accuracy is presented in Table 5.

The statistical performances of these algorithms were measured comparatively by using several goodness-of-fit criteria, namely the coefficient of determination ( $R^{2}=0.662$ , 0.792, 0.624, 0.722, 0.699, and 0.699), adjusted coefficient of determination (Adj. $R^{2}=0.637$ , 0.768, 0.609, 0.721, 0.633, and 0.633), mean square error (MSE = 4.461, 2.277, 4.121, 2.904, 3.296, and 3.296), root mean square error (RMSE = 2.112, 1.509, 2.030, 1.704, 1.815, and 1.815), mean absolute error (MAE = 1.702, 1.193, 1.628, 1.279, 1409, and 1.409) and mean absolute percentage error (MAPE = 4.002, 3.335, 3.967, 3.578, 3.925, and 3.925), between the observed and predicted values of live body weight, respectively. The results of the predictive performances displayed that the MARS data mining algorithm was more informative in the prediction of body weight in Kıvırcık lambs (Table 5).

**Table 5:** Predictive model performance results of goodness-of-fit criteria of data mining algorithms for SW trait.

Method	$R^{2}$	Adj. $R^{2}$	MSE	RMSE	MAE	MAPE
RF	0.662	0.637	4.461	2.112	1.702	4.002
MARS	0.792	0.768	2.277	1.509	1.193	3.335
Bagging MARS	0.624	0.609	4.121	2.030	1.628	3.967
CART	0.722	0.721	2.904	1.704	1.279	3.578
CHAID	0.699	0.633	3.296	1.815	1.409	3.925
Exhaustive CHAID	0.699	0.633	3.296	1.815	1.409	3.925

**Figure 1:**
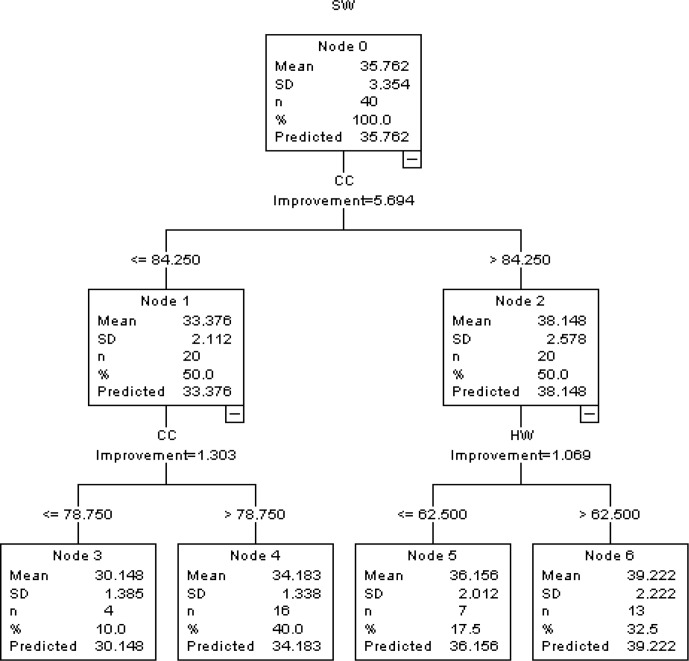
The regression tree diagram constructed by the CART algorithm (results for CART analysis).

The diagram of the CART algorithm applied to determine body characteristics affecting live weight is presented in Fig. 1. For the CART algorithm, the number of parent and child nodes is set to be 10:5 .

In the CART algorithm, the heaviest average body weight was obtained for male Kıvırcık lambs (39.222 kg) with CC > 84.250 cm and HW > 62.500 cm. The lowest average live weight (30.148 kg) was obtained for lambs whose chest circumference was 78.750 cm or less.

**Figure 2:**
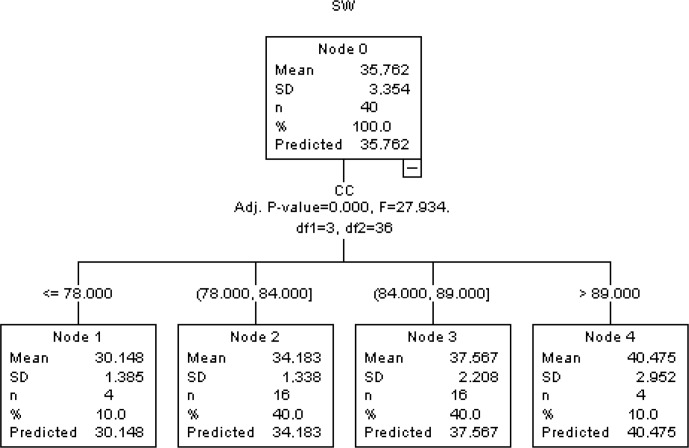
The regression tree diagram constructed by the CHAID algorithm (results for CHAID analysis).

The diagram of the CHAID algorithm applied to determine the body characteristics affecting live weight is presented in Fig. 2. For the CHAID algorithm, the number of parent and child nodes is set to be 10:5 .

As seen in Fig. 2, as a result of the CHAID algorithm, SW was measured to be 30.148 kg when CC ≤ 78, 34.183 kg when 78< CC ≤ 84, 37.567 kg when 84< CC ≤89 , and 40.475 kg when CC >89 . As the CC value increased, the SW value also increased.

**Figure 3:**
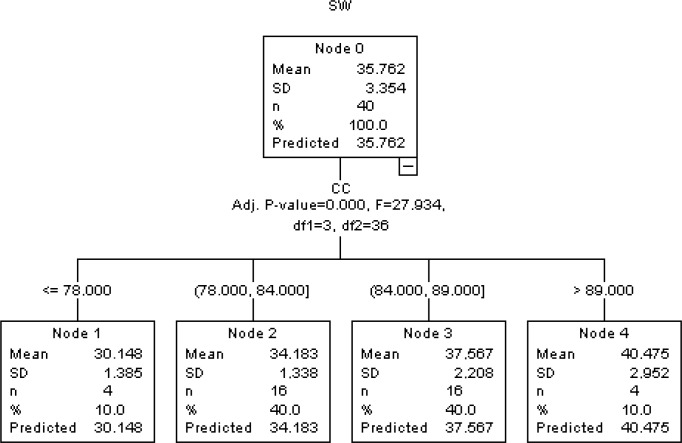
The regression tree diagram constructed by the exhaustive CHAD algorithm (results for exhaustive CHAID analysis).

The diagram of the exhaustive CHAID algorithm applied to determine body measurements affecting live weight is presented in Fig. 3. For the exhaustive CHAID algorithm, the number of parent and child nodes is set to be 10:5 .

As can be seen in Fig. 3, according to the results of the exhaustive CHAID algorithm, if CC ≤ 78 then SW is measured to be 30.148 kg. If 78< CC ≤ 84 then SW is measured to be 34.183. If 84< CC ≤ 89 then SW is measured to be 37.567, and if CC >89 then SW is measured to be 40.475 kg. As the CC value increases, the SW value also increases. As can be seen in both Figs. 2 and 3, the CHAID and exhaustive CHAID algorithms yield the same results. In both the CHAID and exhaustive CHAID algorithms, when CC >89 , the highest final live weight value was estimated to be 40.475 kg.

The optimal MARS predictive model produced the smallest cross-validated RMSE value with four terms and a degree of 1, which means that no interaction effect was used in the model. Also, a cross-validation $R^{2}$ value of 0.710 and an $R^{2}$ value of 0.792 mean that there was no overfitting problem in the constructed MARS model that reflected main characteristics of Kıvırcık lambs.

The prediction equation produced by the MARS algorithm is given below.

13 $$\begin{array}{rl} \mathrm{SW} & =34.43027+0.63405\cdot \text{max}(0,\mathrm{CH}-62.5)\\ & +1.509903\cdot \text{max}(0,\mathrm{CW}-15.5)-0.5107263\\ & \cdot \text{max}(0,85-\text{CC}) \end{array}$$

As a result of the MARS algorithm, if CH >62.5 cm then SW increases by 0.634 kg. If CW >15.5 cm then SW increases by 1.51 kg. If CC ≤ 85 cm then SW decreases by 0.51 kg.

The prediction equation obtained by the MARS algorithm obviously revealed independent-variable effects among significant predictors entered into the MARS model. This case might present a new perspective for lambs breeders compared with the results of earlier studies conducted for the prediction of live body weight. We can predicted the SW (kg) of a Kıvırcık lamb with HW of 70 cm, BH of 69 cm, CH of 69 cm, CD of 30.5, BL of 68 cm, CW of 16 cm, CC of 84 cm, and CWT of 16 cmas follows:

14 $$\begin{array}{rl} \mathrm{SW} & =34.43027+0.63405\cdot \text{max}(0,69-62.5)\\ & +1.509903\cdot \text{max}(0,16-15.5)-0.5107263\\ & \cdot \text{max}(0,85-84). \end{array}$$

**Figure 4:**
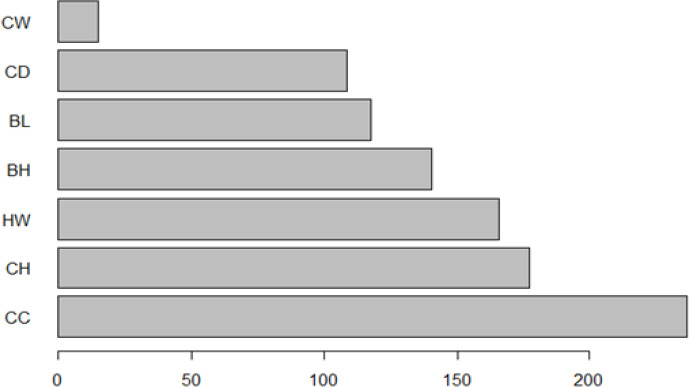
Graph of relative importance of significant independent variables. CC denotes chest circumference (cm), CH denotes croup height (cm), HW denotes height at withers (cm), BH denotes back height (cm), BL denotes body length (cm), CD denotes chest depth (cm), and CW denotes chest width (cm).

According to the above, the predicted SW of the Kıvırcık lamb is 39.817 kg. A graph of relative importance is given in Fig. 4.

The prediction equation produced by the Bagging MARS algorithm is presented in Eq. (A1).

According to the prediction in Eq. (A1) as obtained with the Bagging MARS algorithm in the first bootstrap, the final live weight (SW) demonstrated an increase when HW >62 cm. Likewise, with CH >63 , an incremental increase in the SW of Kıvırcık lambs can be expected. Also, with CD >27 cm, an incremental increase in the SW of lambs would be expected. With CW >15.5 cm, an incremental increase in the SW of lambs would also be anticipated. In the second bootstrap, with CD ≤ 27 cm, an incremental increase in the SW of lambs can be expected. With CW ≤ 15.5 cm, a decline in the SW of lambs can be expected. Similarly, with BL >70 cm, a decline in the SW of lambs can be expected. In the third bootstrap, with CW >15.5 cm, an incremental increase in the SW of lambs can be expected. With BH >59 cm, a reduction in the SW of lambs can be expected. In the fourth bootstrap, with CW >15 cm, an incremental increase in the SW of lambs can be expected. In the fifth bootstrap, with CW >15.5 cm, an incremental increase in the SW of lambs may be anticipated.

**Figure 5:**
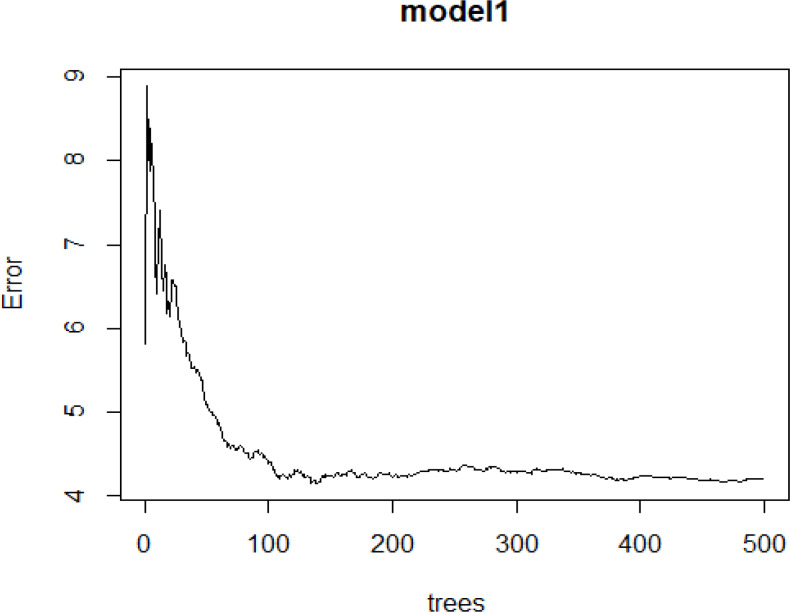
RF algorithm error rate of the model (graph for MSE values according to the number of trees).

In the random forest (RF) application, the dataset was primarily divided into 70 % training and 25 % test data. The HW, BH, CH, CD, BL, CW, and CC variables were given as inputs, and body weight (SW) was estimated. When the seven independent variables in the dataset were given as inputs, the rate of explaining the variance with the random forest algorithm in estimating the live weight was found to be 66.24 %. The mean square error (MSE) graph obtained according to the number of trees in the RF model is shown in Fig. 5.

**Figure 6:**
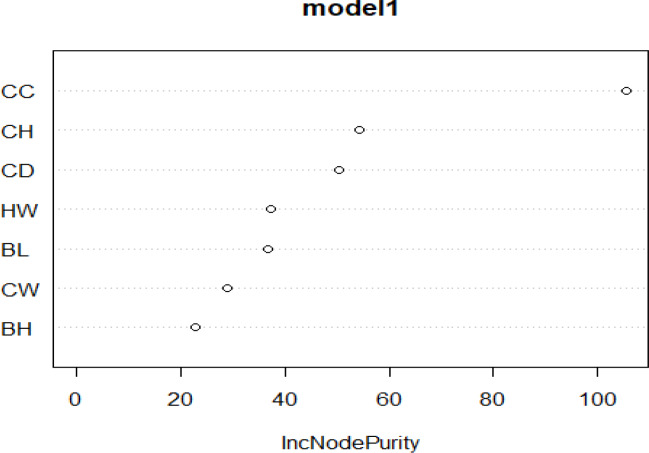
Significance graph of the predictor variables of the RF model.

The significance level of the independent variables in the RF model is presented in Table 6 and Fig. 6.

**Table 6:** Significance level of the independent variables (importance score of predictor variables).

Inc. node	Significance level
HW	37.43347
BH	22.73452
CH	54.13794
CD	50.41466
BL	36.76327
CW	29.07164
CC	105.57146

The MARS algorithm with the highest $R^{2}$ and Adj. $R^{2}$ and the lowest MSE, RMSE, MAE, and MAPE values gave the best results.

Table 6 and Fig. 6 indicate the importance of predictors identified by the RF method for describing the body weight of Kıvırcık lambs. The most important variable in relation to the weight of the animals was found to be the chest circumference (CC). The second-most important variable was the croup height (CH), and the variable of lowest importance was back height (BH). The significance level of the variables is listed as CC > CH > CD > HW > BL > CW > BH.

**Figure 7:**
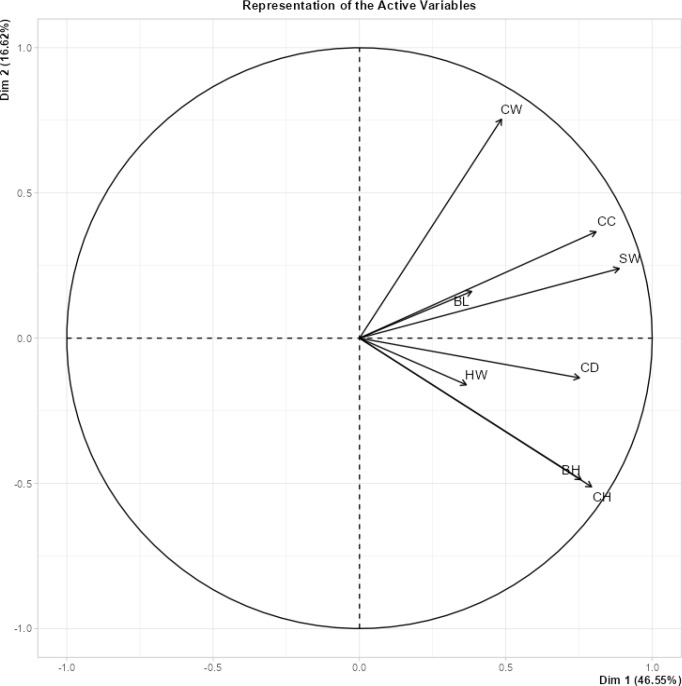
Principal component analysis applied to selected morphological characteristic indicators of the body.

A principal component analysis (PCA) was applied in order to represent the variability of the selected animal-based indicators (BL, CW, CC, SW, CD, HW, BH, and CH) of the 40 Kıvırcık lambs (Fig. 7). As a result of the PCA analysis, PC1 (first major component) was 46.55 %, PC2 (second major component) was 16.62 %, and there was a variation of 63.17 % in total. All variables are in the same direction, and the correlations between them are positive. Since the CW, CC, SW, BH, and CH variables are close to the circle, they are the variables that contribute greatly and show higher variation. The BL and HW variables made smaller contributions as they were far from the circle. The correlation coefficients between BL and all other variables and between HW and all other variables are low. The correlations between BH and CH, BH and CD, CH and CD, CW and CC, CW and SW, CC and SW, CH and SW, and CD and SW were found to be high.

## 5 Discussion

In a previous study, the Pearson correlation coefficients ( r ) between live weight and wither height (for males, pregnant females, and non-pregnant females) were positive and ranged from 0.21 to 0.78 (Kunene et al., 2009). These values are higher than the values obtained in this study for the same variables. Slaughter weights of 42.2, 42.5, and 40.7 kg were found in 6325 crossbred lambs sired by Charollais, Suffolk, and Texel rams in England, Scotland, and Wales, respectively (Márquez et al., 2015). These weights are higher than the slaughter weights of the lambs in this study. This difference may be caused by different growing conditions and the difference in terms of breed. Karabacak et al. (2017) used the CART algorithm for body weight estimation in sheep and found $R^{2}=0.569$ , Adj. $R^{2}=0.549$ , an SD ratio of 0.657, MAE = 0.065, and RMSE = 1.306. In terms of goodness-of-fit statistics, these results differ from the results of the present study. Abbas et al. (2021), in estimating the body weight of Thalli sheep, found that the $R^{2}$ (%) values ranged from 49.28 (CART) to 64.48 (CHAID). The lowest RMSE was found with CHAID (2.61), and the highest was found with CART (3.12). The most significant predictors of live body weight for all algorithms was heart girth. The greatest average body weight (41.12 kg) was noticed in the subgroup of sheep with a body length of >73.91 cm. The CART method used in this study gave better results. In addition, the best predictor for body weight in this study is chest circumference as opposed to heart circumference, as found in previous studies. In the study of Huma and Iqbal (2019), linear model, regression tree, random forest, and support vector machine methods were applied to estimate the body weight of Baluchi sheep. The random forest (RF) method performed much better in estimating body weight. The values of r (0.957) and $R^{2}$ (0.916) were both found to be the highest, while the values of MAE (3.275), RMSE (5.390), and MAPE (7.946) were the lowest for this machine learning method. In this study, the RF method was the third-best method after the MARS and CART methods in estimating the live weight of lambs. Moreover, the most important variable determining body weight according to the RF method is chest circumference, and this is different from the results obtained in previous studies. While the body length of Kıvırcık lambs was higher than that of Baluchi sheep, the wither height and body weight were slightly lower. In Çakmakçi's (2022) study, the acquired results showed that the estimation accuracy validated using the test dataset displayed that the random forest method outperformed all other methods (support vector machines with a radial basis function kernel, classification and regression trees (CART), random forest (RF), and model average neural networks), with the lowest values in terms of MAE, RMSE, and MAPE. Even though the CART algorithm took substantially less time to train, it was the worst-performing method, with the highest values in terms of MAE, RMSE, and MAPE among the methods. In addition, the mean body weight of 93 sheep was 54.7 kg, the mean wither height was 72.9 cm, the body length was 66.7 cm, the mean chest depth was 33.2 cm, the mean chest width was 21.1 cm, the mean rump height was 71.1 cm, and the mean chest circumference was 93 cm. The body length was close to the value found in this study, but the values of other body characteristics were higher than the values found in this study. This difference is due to the different breeds and ages of the animals.

In another study (Ali et al., 2015), the coefficients of determination ( $R^{2}$ %) between the actual and predicted body weight values in Harnai sheep for the CHAID, exhaustive CHAID, CART, and ANN algorithms were found to be 83.770 %, 84.210 %, 82.644 %, and 81.999 % respectively; the adjusted coefficients of determination were 83.354 %, 83.805 %, 82.199 %, and 81.537 %, respectively; and the estimates of the root mean square error were 1.509, 1.488, 1.560, and 1.589, respectively. All of the analyzed algorithms gave very similar results, but the results of the quality criteria displayed that the exhaustive CHAID algorithm was better than the CHAID, CART, and ANN algorithms because the exhaustive CHAID obtained the highest r , $R^{2}$ (%), and Adj. $R^{2}$ values and the lowest RMSE statistics, indicating good performance (Ali et al., 2015). When evaluated in terms of model performance, the results of the exhaustive CHAID algorithm as used in the study of Ali et a. (2015) differed from the results of this study.

## 6 Conclusions

Based upon the results of the existing study, it was finalized that the MARS algorithm was more influential for live-weight determination in Kıvırcık lambs based on the measurement of body characteristics, as illustrated by its lower error measurements compared to the Bagging MARS, CART, CHAID, exhaustive CHAID, and RF algorithms. Using the CART algorithm, the highest body weight was reached in lambs with CC > 84.25 and HW > 62.5 (39.222 kg). Using the RF algorithm, the variables affecting body weight the most were CC, CH, and CD. According to the Bagging MARS algorithm, while, in first bootstrap, the variables that contributed the most positively to live weight were HW > 62 cm (3.47 kg increase) and CD > 27 cm (3.20 kg increase), the variables that contributed the most negatively were HW ≤ 63 cm (4.28 kg decrease). According to the MARS algorithm, if CH > 62.5 cm and CW > 15.5 cm then SW increases by 0.634 and 1.51 kg, respectively. However, if CC ≤ 85 cm then SW decreases by 0.51 kg. The three most important variables affecting SW were CC, CH, and HW. These findings are likely to support sheep farming researchers and producers in choosing the best predictors to increase live weight in lambs by means of the selection of high-performance genotypes.

In general, according to the results obtained from correlation coefficient, principal component analysis, and data mining methods, the most important body measurement affecting live weight in lambs is chest circumference.

## Data Availability

The data are accessible from the contact author upon request.

## Appendix A

A1
$$\begin{array}{rl} & \text{SW}=(31.05119)+3.468963\cdot \text{h(HW-62)}-4.281747\\ & \;\cdot \text{h(HW-63)}+0.302435\cdot \text{h(63-CH)}+1.685739\\ & \;\cdot \text{h(CH-63)}-1.633999\cdot \text{h(CH-64)}+1.555153\cdot \text{h(27-CD)}\\ & \;+3.204299\cdot \text{h(CD-27)}-3.946664\cdot \text{h(CD-28.5)}\\ & \;-0.04111699\cdot \text{h(68.5-BL)}+1.148383\cdot \text{h(CW-15.5)}\\ & \;-0.4592397\cdot \text{h(85-CC)}+38.18995+0.02376369\\ & \;\cdot \text{h(65-HW)}-0.843566\cdot \text{h(BH-59.5)}-0.9992979\\ & \;\cdot \text{h(62-BH)}+0.9767741\cdot \text{h(63-CH)}+0.4819635\\ & \;\cdot \text{h(CH-63)}+1.856435\cdot \text{h(27-CD)}+1.651628\cdot \text{h(CD-27)}\\ & \;-0.7452372\cdot \text{h(70-BL)}-1.244336\cdot \text{h(BL-70)}-6.855966\\ & \;\cdot \text{h(15.5-CW)}+0.2925685\cdot \text{h(CC-83)}+33.44702\\ & \;+0.6281928\cdot \text{h(BH-57)}-0.7244647\cdot \text{h(BH-59)}\\ & \;+0.8205228\cdot \text{h(CH-65)}+0.9376569\cdot \text{h(27-CD)}\\ & \;+0.6857602\cdot \text{h(CD-27)}+2.751479\cdot \text{h(CW-15.5)}\\ & \;-0.5415199\cdot \text{h(85-CC)}-0.323558\cdot \text{h(CC-85)}+33.34886\\ & \;-0.8182725\cdot \text{h(HW-64)}+0.8266158\cdot \text{h(57-BH)}\\ & \;-0.682322\cdot \text{h(BH-57)}+2.479122\cdot \text{h(CH-65.5)}\\ & \;+0.8352139\cdot \text{h(27.5-CD)}-0.814892\cdot \text{h(67.5-BL)}\\ & \;-0.9692761\cdot \text{h(BL-67.5)}+2.5073\cdot \text{h(BL-69)}+3.353495\\ & \;\cdot \text{h(15-CW)}+5.292117\cdot \text{h(CW-15)}-6.099768\cdot \text{h(CW-16)}\\ & \;-1.040533\cdot \text{h(80-CC)}-0.5825832\cdot \text{h(CC-80)}+1.432153\\ & \;\cdot \text{h(CC-85.5)}+32.6558+0.767535\cdot \text{h(CH-63)}\\ & \;+0.7462429\cdot \text{h(CD-27)}+1.847943\cdot \text{h(CW-15.5)}\\ & \;-0.4186789\cdot \text{h(82-CC)})/5 \end{array}$$

## Code availability

All codes utilized during this study are included in this published article. It was implemented in the R Studio program (R Core Team, 2020).
